# Hospitalizations for Community-Acquired and Non-Ventilator-Associated Hospital-Acquired Pneumonia in Spain: Influence of the Presence of Bronchiectasis. A Retrospective Database Study

**DOI:** 10.3390/jcm9082339

**Published:** 2020-07-22

**Authors:** Gema Sanchez-Muñoz, Ana López-de-Andrés, Valentín Hernández-Barrera, Fernando Pedraza-Serrano, Rodrigo Jimenez-Garcia, Marta Lopez-Herranz, Luis Puente-Maestu, Javier de Miguel-Diez

**Affiliations:** 1 Respiratory Department, Hospital General Universitario Gregorio Marañón, Facultad de Medicina, Universidad Complutense de Madrid, Instituto de Investigación Sanitaria Gregorio Marañón (IiSGM), 28009 Madrid, Spain; gesam76@yahoo.es (G.S.-M.); fpedrazaserrano@gmail.com (F.P.-S.); luis.puente@salud.madrid.org (L.P.-M.); javier.miguel@salud.madrid.org (J.d.M.-D.); 2 Preventive Medicine and Public Health Teaching and Research Unit, Health Sciences Faculty, Rey Juan Carlos University, Alcorcón, 28922 Madrid, Spain; valentin.hernandez@urjc.es; 3 Department of Public Health & Maternal and Child Health, Faculty of Medicine, Universidad Complutense de Madrid, 28040 Madrid, Spain; rodrijim@ucm.es; 4 Faculty of Nursing, Physiotherapy and Podology, Universidad Complutense de Madrid, 28040 Madrid, Spain; martal11@ucm.es

**Keywords:** community-acquired pneumonia, non-ventilator hospital-acquired pneumonia, hospitalizations, bronchiectasis, in-hospital mortality

## Abstract

To examine and compare in-hospital mortality (IHM) of community-acquired pneumonia (CAP) and non-ventilator hospital-acquired pneumonia (NV-HAP) among patients with or without bronchiectasis (BQ) using propensity score matching. A retrospective observational epidemiological study using the Spanish Hospital Discharge Records, 2016–17. We identified 257,455 admissions with CAP (3.97% with BQ) and 17,069 with NV-HAP (2.07% with BQ). Patients with CAP and BQ had less comorbidity, lower IHM, and a longer mean length of hospital stay (*p* < 0.001) than non-BQ patients. They had a higher number of isolated microorganisms, including *Pseudomonas aeruginosa*. In patients with BQ and NV-HAP, no differences were observed with respect to comorbidity, in-hospital mortality (IHM), or mean length of stay. *P. aeruginosa* was more frequent (*p* = 0.028). IHM for CAP and NV-HAP with BQ was 7.89% and 20.06%, respectively. The factors associated with IHM in CAP with BQ were age, comorbidity, pressure ulcers, surgery, dialysis, and invasive ventilation, whereas in NV-HAP with BQ, the determinants were age, metastatic cancer, need for dialysis, and invasive ventilation. Patients with CAP and BQ have less comorbidity, lower IHM and a longer mean length of hospital stay than non-BQ patients. However, they had a higher number of isolated microorganisms, including *Pseudomonas aeruginosa*. In patients with BQ and NV-HAP, no differences were observed with respect to comorbidity, in-hospital mortality, or mean length of stay, but they had a greater frequency of infection by *P. aeruginosa* than non-BQ patients. Predictors of IHM for both types of pneumonia among BQ patients included dialysis and invasive ventilation.

## 1. Introduction

Bronchiectasis (BQ) is a disease characterized by abnormal and irreversible dilations of the bronchi, with alteration of the ciliary epithelium and secondary symptoms [1]. It generally manifests as a cough, expectoration, and persistent or recurrent respiratory infections [2,3,4]. The overall perception and management of BQ has varied dramatically in recent years, and the disease has become increasingly relevant owing to its greater prevalence and the negative impact of its co-occurrence with other diseases. BQ is currently considered the third most common chronic inflammatory disease of the airway after asthma and chronic obstructive pulmonary disease [2,3]. However, the real prevalence of this condition remains unknown, although it is thought to range between 53 and 566 cases per 100,000 inhabitants. BQ more commonly affects women and older people [4,5,6].

In most series analyzed, respiratory infections continue to be the main cause of BQ [7,8,9]. Furthermore, the presence of BQ favors the development of respiratory infections, including pneumonia, and generates considerable health care costs [10]. Nevertheless, very few studies have evaluated the prevalence and characteristics of community-acquired pneumonia (CAP) in patients with BQ [11,12,13,14], and even fewer have examined non-ventilator hospital-acquired pneumonia (NV-HAP) [15]. Furthermore, when the diagnosis of BQ is delayed, we often discover numerous previous episodes of respiratory infection or pneumonia, with the consequent impairment of lung function and quality of life. BQ increases the risk of infection by *Pseudomonas aeruginosa* and other pathogens that are resistant to initial empirical treatment of pneumonia. Thus, BQ is a risk factor for the emergence of resistance to antibiotics in the treatment of pneumonia [16,17,18].

Important epidemiological information on BQ can be obtained using hospital discharge databases. Collecting data on admissions for pneumonia in patients with BQ at a national level can shed light on the incidence, patient characteristics, mean length of hospital stay, and in-hospital mortality (IHM). Furthermore, analysis of the trends and characteristics of hospitalizations for this subgroup at a national level and comparison with results from other countries can improve our knowledge and management of this disease.

In this study, our objectives were as follows: (i) to examine the characteristics of CAP and NV-HAP among patients with and without BQ in Spain during the period 2016–17; (ii) to compare IHM for CAP and NV-HAP between patients with and without BQ using propensity score matching (PSM); and (iii) to identify factors associated with IHM after CAP and NV-HAP among patients with BQ.

## 2. Materials and Methods

### 2.1. Design, Setting, and Participants

This observational retrospective epidemiological study was conducted using the Hospital Discharge Records of the Spanish National Health System (RAE-CMBD, Registro de Actividad de Atención Especializada-Conjunto Mínimo Básico de Datos (Register of Specialized Care Activity-Basic Minimum Database)) from 1 January 2016 to 31 December 2017. The RAE-CMBD provides anonymous detailed medical information on over 92% of admissions to Spanish public and private hospitals, including discharge diagnoses (up to 20) and procedures performed (up to 20) during the hospital stay using the codes of the International Classification of Disease, 10th Revision (ICD-10) [19]. Each discharge diagnosis has a “Present on Admission” (POA) indicator assigned according to the ICD-10-CM Official Guidelines for Coding and Reporting (https://icdlist.com/icd-10/guidelines/). The reporting options and definitions for POA are “Y” (present at the time of inpatient admission), “N” (not present at the time of inpatient admission), “U” (documentation is insufficient to determine if the condition was present at the time of inpatient admission), “W” (provider is unable to clinically determine whether condition was present at the time of inpatient admission), and “1” (unreported/not used. Exempt from POA reporting).

The study population comprised all admissions of patients who were hospitalized with a diagnosis of pneumonia. We defined CAP as any hospitalization that included any of the following conditions: (i) any ICD-10 code from J12 to J18 as the primary diagnosis with a POA indicator of “Y”; and (ii) any ICD-10 code from J12 to J18 in any of the secondary diagnosis fields (2–20) and with a POA indicator of “Y”. NV-HAP is defined as an episode of pneumonia unassociated with mechanical ventilation that is not incubating at the time of admission to hospital. We identified NV-HAP in patients with any ICD-10 codes from J12 to J18 in any diagnosis position and with a POA indicator coded as “N” that had been hospitalized for ≥48 h. We excluded hospitalizations with ventilator-associated pneumonia defined as any hospitalization with diagnostic ICD-10 code J95.851 in any position, influenza-related pneumonia (ICD-10 codes: J09, J10, J11), aspiration pneumonia (J69, J69.0, J69.1, J69.8), and hospitalizations with ICD-10 codes from J12 to J18 in any diagnosis fields and with a POA indicator coded as “U” or “W” or “unreported/not used”.

We grouped admissions by BQ status as follows: “BQ patients” if the ICD-10 code J47.x was recorded in any position (1–20) and with a POA indicator of “Y” and “non-BQ patients” if no codes for BQ appeared in any diagnostic position. Thus, any patient that was diagnosed with BQ during the admission (POA indicator of “N”) would not be included in our investigation.

### 2.2. Main Outcome Measures

Our main outcome measure is the IHM after CAP and NV-HAP among patients with and without BQ. Secondarily, we compared covariates such as demographic information (age and sex), diagnosed comorbidities, therapeutic procedures, and length of hospital stay (LOHS) according to the presence of BQ.

### 2.3. Study Variables

To assess the burden of comorbidity, all conditions included in the Charlson Comorbidity Index (CCI) coded in any diagnosis position in the discharge report were identified [20]. The ICD-10 codes used to identify the conditions of the CCI are those described by Quan et al. [21].

The RAE-CMBD includes a variable with the diagnosis-related groups categorized as medical/surgical/other. This was used to identify those patients who underwent any type of surgical procedure during their hospital admission [19].

Irrespective of the position on the procedure coding list, we specifically identified the following procedures: computed axial tomography of the thorax, fiberoptic bronchoscopy, non-invasive mechanical ventilation, invasive mechanical ventilation, and dialysis. The diagnosis of pressure ulcer was also identified. We analyzed pressure ulcers because previous investigations have associated the occurrence of pneumonia and its outcome with pressure ulcers [22,23]. Furthermore, in our opinion, the diagnosis of pressure ulcers can influence the morbidity and mortality of patients with BQ and the length of hospital stay. The ICD-10 codes used for this purpose are shown in Table S1.

We analyzed pathogens documented during hospitalizations for pneumonia using the following ICD-10 codes: A48.1 for *Legionella*; B37.1 for candidiasis; B44.9 for *Aspergillus*; J13 for *Streptococcus pneumoniae*; J14 for *Haemophilus influenzae*; J15 for *Klebsiella pneumoniae*; J15.1 for *Pseudomona aeruginosa*; J15.211 and J15.212 for *Staphylococcus aureus*; J15.4 for pneumonia due to other streptococci; J15.5 for *Escherichia coli*; and J15.6 for other Gram-negative bacteria.

### 2.4. PSM Method

We used PSM to obtain unbiased matched populations of BQ and non-BQ patients adjusted for the impact of previous confounding factors [24]. PSM consists of selecting BQ patients and non-BQ patients with the same, or nearly the same, propensity score obtained using logistic regression in order to match the structure of confounding factors for both groups of patients [24,25]. The variables included in the PSM model were sex, age, all comorbidities analyzed, and whether the patient underwent surgery. Historically, PSM is typically used to "allow investigators to estimate causal treatment effects using observational or nonrandomized data”. However, as described by Szklo M. and Nieto F.J., PSM is a method that can be used to mimic randomization by making the exposed and non-exposed cohort as comparable as possible with respect to relevant confounding variables [26]. In our investigation, the exposed cohort makes up those suffering BQ and the non-exposed cohort those not suffering this condition. Therefore, propensity scores can be thought of as an advanced matching technique that makes possible the comparison of populations with very different sizes and distributions according to possible confounding variables [26]. This approach, considering exposed and non-exposed patients or those without a condition and not a treatment or an intervention, has been previously used by other authors [27,28,29,30,31].

### 2.5. Statistical Methods

A descriptive statistical analysis was performed for all continuous variables and categories. Variables are expressed as percentages and as means/medians with standard deviations/interquartile ranges. To assess differences between BQ and non-BQ patients, the statistical tests conducted for continuous variables were the t-test for normal distributions and the Mann–Whitney test for non-normal distributions; categorical variables were compared using the chi-square test.

The paired t-test was used for continuous variables and the McNemar test was used for categorical variables to compare BQ and non-BQ patients after matching [32].

In order to identify variables associated with IHM as a binary outcome among BQ patients with both types of pneumonia, we performed multivariable logistic regression analyses. The variables included in the models were those with significant results in the bivariable analysis and those considered relevant in other investigations [10,11,12,13,14,15]. The estimate applied was the odds ratio (OR) with its 95% CI.

In order to check the validity of the PSM, and as a sensitivity analysis, we also built a multivariable logistic regression model using the entire database to assess the effects of BQ on the IHM among patients with CAP and NV-HAP after controlling for possible confounders.

PSM and all statistical analysis were performed using Stata version 10.1 (Stata, College Station, Texas, USA). Statistical significance was set at *p* < 0.05 (2-tailed).

### 2.6. Ethical Aspects

The RAE-CMBD is owned by the Spanish Ministry of Health, which provided us with the database. When we received the database, all personal identifiers had been deleted to guarantee data confidentiality. According to Spanish legislation and given the type of data used in our investigation, it was not necessary to obtain the approval of an ethics committee.

## 3. Results

A total of 274,524 patients were hospitalized with a diagnosis of CAP or NV-VAP in Spain during the period 2016–2017. CAP was diagnosed in 257,455 cases (patients with BQ, 3.97%) and NV-HAP in 17,069 (patients with BQ, 2.07%).

### 3.1. Clinical Characteristics and in-Hospital Outcomes of Patients Hospitalized with CAP and NV-HAP According to BQ Status

CAP was identified more frequently among men than women in both groups (60.65% and 58.89% for BQ and non-BQ patients, respectively; *p* < 0.001). Overall, patients with BQ were significantly older (75.47 years; SD = 13.67) than patients without BQ (68.63 years; SD = 24.47) and had significantly fewer co-existing medical conditions (*p* < 0.001). Specifically, lower prevalence was recorded for acute myocardial infarction (3.55% vs. 4.18%; *p* = 0.002), congestive heart failure (18.4% vs. 19.97%; *p* < 0.001), cerebrovascular disease (5% vs. 6%; *p* < 0.001), type 2 diabetes mellitus (23.57% vs. 24.59%; *p* = 0.019), and cancer (7.17% vs. 8.23%; *p* < 0.001). In the case of dementia, hemiplegia or paraplegia, and metastatic cancer, prevalence was two times lower (all *p* values < 0.001). However, the prevalence of peripheral vascular disease, rheumatoid disease, and mild liver disease was significantly higher in BQ patients (*p* < 0.001). A significantly lower percentage of BQ patients underwent surgery (2.09%) compared to non-BQ patients (3.2%). Mean LOHS was higher in BQ patients (10.44 days vs. 9.30 days; *p* < 0.001). Crude IHM was significantly lower for BQ patients than for non-BQ patients (7.89% vs. 11.81%; *p* < 0.001) (Table 1).

NV-HAP was identified more frequently among men than among women in both populations studied (68.36% and 63.31% for BQ and non-BQ patients, respectively; *p* = 0.049), and mean age was significantly higher in those with BQ (72.44 vs. 65.83 years; *p* < 0.001). Prevalence values were higher in BQ patients for congestive heart failure (29.66% vs. 22.43%; *p* = 0.001), rheumatoid disease (4.24% vs. 1.93%; *p* = 0.002), and mild liver disease (7.63% vs. 5%; *p* = 0.025), although they had a lower prevalence of cerebrovascular disease (9.89% vs.13.53%; *p* = 0.047) and hemiplegia or paraplegia (1.69% vs. 4.88%; *p* = 0.006). We found that BQ patients had undergone surgery significantly more frequently than non-BQ patients (28.25% vs. 41.66%; *p* < 0.001). Crude IHM was 20.06% for BQ patients and 25.63% for non-BQ patients (*p* = 0.017) (Table 1).

### 3.2. Distribution of Study Covariates Among Bronchiectasis and Non-Bronchiectasis Patients Hospitalized with CAP and NV-HAP After PSM

Table 2 shows the characteristics of patients admitted with CAP and BQ and control patients (non-BQ) after PSM. In patients with BQ, significantly higher frequencies were recorded for computed axial tomography of the thorax (11.71% vs. 6.66%; *p* < 0.001), fiberoptic bronchoscopy (1.4% vs. 0.99%; *p* = 0.007), and non-invasive mechanical ventilation (2.42% vs. 1.94%; *p* = 0.017). However, BQ patients had lower rates of invasive mechanical ventilation (1.47% vs. 2%; *p* = 0.003) and pressure ulcers (1.22% vs. 2.24%; *p* < 0.001).

The mean LOHS was 10.44 ± 9.64 days among patients with BQ and 8.96 ± 8.52 days among matched controls (*p* < 0.001). After PSM, the IHM during admission for CAP was 7.89% in patients with BQ and 10.67% in matched controls (*p* < 0.001) (Table 2).

Table S2 shows that the prevalence of all pneumonia pathogens in CAP, except for unspecified *Streptococcus* species, was significantly higher among BQ patients after PSM. The most frequently isolated microorganism was *Streptococcus pneumoniae*.

Comparison of BQ patients with matched controls who had an episode of NV-HAP after PSM (Table 3) revealed higher frequencies of computed axial tomography of the thorax (0.17% vs. 0.1%; *p* = 0.011). No significant differences were found regarding LOHS (27.05 days vs. 29.58 days; *p* = 0.321) or IHM (20.06% vs. 25.42% *p* = 0.088).

As shown in Table S2, the prevalence of *P. aeruginosa* was higher among BQ patients with NV-VAP than among non-BQ patients (0.07% vs. 0.03%; *p* = 0.028).

### 3.3. Multivariable Logistic Regression Analysis of the Factors Associated with IHM Among Bronchiectasis Patients

Table 4 shows the result of the multivariable logistic regression analysis of the factors independently associated with IHM after CAP and NV-HAP among bronchiectasis patients.

Older age was associated with IHM in patients with CAP (vs. <40 years old, OR, 2.82; 95% CI, 1.01-7.89 for 65–74 years and OR, 6.98; 95% CI, 2.53–19.25 for ≥75 years). However, older age was associated with lower mortality in patients with NV-VAP (vs. <40 years old; OR, 0.22; 95% CI, 0.08–0.64).

The presence of metastatic cancer increased the probability of death in patients with CAP and NV-HAP. Invasive mechanical ventilation and dialysis were also associated with IHM in patients with CAP and NV-HAP.

In patients with CAP, the risk of death increased with the presence of congestive heart failure, dementia, cancer, and moderate or severe liver disease. Furthermore, patients with pressure ulcers who had undergone surgery had a high risk of IHM. However, patients with a code for *S. pneumoniae* and *H. influenzae* were also associated with lower mortality.

### 3.4. Sensitivity Analysis

Shown in Table S3 are the results of the multivariable logistic regression model using the entire database to assess the effects of BQ on the IHM among patients with CAP and NV-HAP. As can be seen, patients with a diagnosis of BQ and CAP had a lower probability of dying during their hospitalization than patients without BQ (OR 0.88; 95% CI 0.81–0.95).

## 4. Discussion

In the present study, we observed BQ in 3.97% of patients admitted with CAP and in 2.07% of those admitted with NV-HAP. However, the real prevalence of BQ in patients with pneumonia has not been studied in depth, and the results obtained to date are disparate, ranging from 3% to 24.6%, depending on the series analyzed [12,14,33,34,35]. These differences in the results can be explained in part by differences in method, patient characteristics, and diagnostic criteria. Polverino et al. [14] studied 3731 patients with CAP and reported BQ in 124 cases, with the diagnosis confirmed by high-resolution computed tomography in 111 (3%). In this study, as in ours, BQ patients were older, although, in contrast to our findings, CAP associated with BQ was more common in women and patients with more comorbid conditions. These discrepant results could be due to differences in the design of each study; therefore, it would be interesting to have wider-ranging studies based on similar methods that would enable us to analyze the epidemiological characteristics of patients and to compare them with data obtained in other countries.

The mean length of hospital stay of patients admitted with CAP was significantly greater in those who had BQ than in those who did not. BQ has been considered a risk factor for the presence of pathogens that are potentially resistant to antibiotics, such as *P. aeruginosa* [16,17,18]. The presence of BQ could lead to more frequent failures of initial empirical antibiotic therapy and more days of intravenous treatment, thus increasing hospital stay and associated costs. Regarding this point, a study by Scioscia et al. has seen how the microbiological isolation of *P. aeruginosa* is associated with the need for a longer cycle of antibiotic therapy even during exacerbations of BQ [36].

In-hospital mortality was lower in cases with CAP and BQ even though the patients were older, probably owing to the lower percentage of comorbidities in the group with BQ. No significant differences were observed with respect to comorbidities and IHM in NV-HAP when the presence of BQ was taken into account. However, Parrot et al. [15] found that the mortality of patients with HAP was associated with lung cancer and BQ.

Patients with BQ generally had to undergo more diagnostic procedures (computed tomography and fiberoptic bronchoscopy) than non-BQ patients, for various reasons. First, computed tomography is the diagnostic method of choice for BQ. In addition, the possibility of hemoptysis or processes requiring respiratory samples would probably be greater in these cases than in those where the patient did not have BQ. No differences were detected with respect to invasive ventilation. Non-invasive mechanical ventilation was used more frequently in patients with CAP and BQ, with no differences found in the case of the NV-HAP patients. Admission to the intensive care unit is uncommon in patients with BQ, although data in this area are lacking [37,38]. Nevertheless, the frequency of such admissions is increasing, as reflected in the study by Navaratnam et al. [38], who recorded an annual 8% increase in admissions to the intensive care unit for patients with BQ.

With respect to microbiology results, pathogens were isolated more frequently in patients with CAP and BQ than in those without BQ. The most common causal agent was *S. pneumoniae*, followed by *P. aeruginosa*. These results are similar to those observed in previous studies, such as that by Polverino et al. [14], who evaluated the characteristics of exacerbations of BQ with emphasis on the presence of pneumonia. The authors found that the main causal agents of exacerbations of BQ in patients with and without pneumonia were *S. pneumoniae* and *P. aeruginosa*, respectively. In the case of NV-HAP, *P. aeruginosa*—but not other pathogens—was highly frequent in the group with BQ. *P. aeruginosa* is clearly the most relevant pathogen in patients with BQ of any cause owing to its role in the prognosis of affected patients [39,40,41]. These data reinforce the importance of carrying out appropriate microbiology testing in patients with BQ and pneumonia, irrespective of severity or place of acquisition, in order to identify causal agents that are potentially resistant to the standard antibiotic regimens recommended for these processes and to reduce the number of therapeutic failures. Nevertheless, more studies are needed to better determine optimal diagnostic and treatment procedures in this subgroup of patients.

The determinants of greater mortality in patients with CAP and BQ were age, comorbidity (congestive heart failure, dementia, cancer, liver disease, metastasis, dialysis, and surgery), pressure ulcers, and mechanical ventilation. Isolation of *S. pneumoniae* and *H. influenzae* were considered protective factors. Given that all clinical guidelines and regulations recommend starting empirical treatment that always covers *S. pneumoniae* and *H. influenzae*, the percentage of initial therapeutic failures will probably be smaller when these pathogens are the causal microorganisms. In cases of NV-HAP and BQ, the factors associated with increased IHM were an age range of 65–74 years, dialysis, metastasis, and invasive mechanical ventilation. Few studies evaluate general mortality indices in patients with BQ [32,33]. Particularly noteworthy is the study by Roberts et al. [42], who recorded 5700 deaths from BQ in England and Wales between 2001 and 2007, with an annual 3% increase in mortality owing to BQ. This important finding should lead us to think that the prognosis of BQ is not trivial but that associated mortality could be increasing.

The main strength of our study lies in its large sample size and standardized methodology, which remained constant throughout the study period. Furthermore, we have used a PSM method to make two initially different populations comparable and obtained results similar to those found in a sensitivity analysis using the entire population. Nevertheless, our study is subject to a series of limitations that should be taken into consideration when interpreting the results. First, a potential source of bias lies in the use of ICD-10 codes to identify patients hospitalized for BQ. Unfortunately, the validity of the BQ diagnosis using ICD-10 codes has not been assessed in the RAE-CMBD. Therefore, we are unable to confirm that the diagnosis of BQ in the discharge records is accurate. We did not have access to computerized axial tomography of thorax data and therefore cannot be sure that in each recorded case of BQ the diagnosis was attained according to current guidelines. However, other authors have suggested that as the diagnosis is usually made in a secondary care setting and requires a computerized axial tomography of thorax, it is unlikely that a diagnosis of BQ would be recorded by the discharging physician without confirmation from secondary care. [43,44] Our operational definitions for BQ have been utilized in other published studies [33,45,46,47] but to our knowledge have not been formally validated against a “gold standard” and thus their accuracy is still unknown. Secondly, the RAE-CMBD does not collect information on the type or duration of antibiotic therapy performed, and this certainly determines the patient’s outcome. Thirdly, we did not have data on the severity of the BQ, as this information is not collected in the database, and it can also influence the patient’s outcome. Finally, as we lack results of radiology and other clinical data, it is not possible to distinguish between admissions for CAP/NV-HAP and BQ versus those admitted for BQ exacerbations. However, in the database we have found that the code for BQ exacerbations (J47.1) was present in only 337 (3.29%) of patients with BQ and CAP and in 11 (3.11%) patients with BQ and NV-HAP. Therefore, in our opinion, if there is a codification error between BQ exacerbations and pneumonia, this would be of a small magnitude and unlikely to affect our conclusions. Furthermore, previous studies using administrative data have demonstrated the validity of pneumonia diagnosis when compared with clinical data [48,49].

Despite these limitations, the RAE-CMBD discharge has the advantage of being mandated by the National Public Health System and includes almost 100% of admissions in Spain. In addition, given that Spain is a large country with a public health system providing full, free-of-charge medical services to the entire population, patients come from a variety of socioeconomic categories, thus improving the external validity of the current results.

## 5. Conclusions

Patients with CAP and BQ have less comorbidity, lower IHM, and a longer mean length of stay than non-BQ patients. However, they had a higher number of isolated microorganisms, including *Pseudomonas aeruginosa*. In patients with BQ and NV-HAP, no differences were observed with respect to comorbidity, in-hospital mortality, or mean length of stay, but these patients had a greater frequency of infection by *P. aeruginosa* than non-BQ patients. Predictors of IHM for both types of pneumonia among BQ patients included dialysis and invasive ventilation. Nevertheless, more studies with more accurate methodological approaches will be necessary before we can confirm our findings and further characterize this subgroup of patients.

## Figures and Tables

**Table 1:** Clinical characteristics and in-hospital outcomes of patients hospitalized with community-acquired pneumonia (CAP) and non-ventilator hospital-acquired pneumonia (NV-HAP) in Spain (2016–17) according to the presence of concomitant bronchiectasis.

	Community-Acquired Pneumonia		*p*-Value	Non-Ventilator Hospital-Acquired Pneumonia		*p*-Value
	Bronchiectasis	No Bronchiectasis		Bronchiectasis	No Bronchiectasis	
N	10,230	247,225	NA	354	16,715	NA
Female Sex, *n* (%)	4026 (39.35)	101,645 (41.11)	<0.001	112 (31.64)	6133 (36.69)	0.049
Age, Mean (SD)	75.47 (13.67)	68.63 (24.47)	<0.001	72.44 (18.82)	65.83 (24.18)	<0.001
<40, *n* (%)	263 (2.57)	29,053 (11.75)	<0.001	23 (6.5)	2051 (12.27)	<0.001
40–64, *n* (%)	1369 (13.38)	42,856 (17.33)	<0.001	50 (14.12)	3635 (21.75)	<0.001
65–74, *n* (%)	2054 (20.08)	40,062 (16.2)	<0.001	62 (17.51)	3273 (19.58)	<0.001
≥75, *n* (%)	6544 (63.97)	135,254 (54.71)	<0.001	219 (61.86)	7756 (46.4)	<0.001
Charlson Comorbidity Index, Mean (SD)	1.02 (0.99)	1.09 (1.07)	<0.001	1.35 (1.13)	1.36 (1.15)	0.784
Acute Myocardial Infarction, *n* (%)	363 (3.55)	10,329 (4.18)	0.002	17 (4.8)	1099 (6.57)	0.182
Congestive Heart Failure, *n* (%)	1882 (18.4)	49,361 (19.97)	<0.001	105 (29.66)	3750 (22.43)	0.001
Peripheral Vascular Disease, *n* (%)	678 (6.63)	11,743 (4.75)	<0.001	27 (7.63)	1255 (7.51)	0.933
Cerebrovascular Disease, *n* (%)	512 (5)	14,826 (6)	<0.001	35 (9.89)	2262 (13.53)	0.047
Dementia, *n* (%)	498 (4.87)	21,165 (8.56)	<0.001	17 (4.8)	921 (5.51)	0.563
Rheumatoid Disease, *n* (%)	397 (3.88)	5549 (2.24)	<0.001	15 (4.24)	322 (1.93)	0.002
Peptic Ulcer, *n* (%)	48 (0.47)	1305 (0.53)	0.421	10 (2.82)	298 (1.78)	0.145
Mild Liver Disease, *n* (%)	546 (5.34)	10,736 (4.34)	<0.001	27 (7.63)	835 (5)	0.025
Type 2 Diabetes Mellitus, *n* (%)	2411 (23.57)	60,783 (24.59)	0.019	91 (25.71)	3774 (22.58)	0.164
Hemiplegia or Paraplegia, *n* (%)	46 (0.45)	2042 (0.83)	<0.001	6 (1.69)	815 (4.88)	0.006
Renal Disease, *n* (%)	1618 (15.82)	40,840 (16.52)	0.060	55 (15.54)	2795 (16.72)	0.554
Cancer, *n* (%)	734 (7.17)	20,342 (8.23)	<0.001	35 (9.89)	2660 (15.91)	0.002
Moderate/Severe Liver Disease, *n* (%)	91 (0.89)	2233 (0.9)	0.886	10 (2.82)	520 (3.11)	0.759
Metastatic Cancer, *n* (%)	213 (2.08)	10,836 (4.38)	<0.001	23 (6.5)	1394 (8.34)	0.214
AIDS, *n* (%)	106 (1.04)	2334 (0.94)	0.346	4 (1.13)	105 (0.63)	0.241
Underwent Surgery, *n* (%)	214 (2.09)	7900 (3.2)	<0.001	100 (28.25)	6964 (41.66)	<0.001
Length of Hospital Stay, Mean (SD), (Median/Inter Quartile Range)	10.44 (9.64) (8/7)	9.30 (7.38) (7/6)	<0.001	27.05 (25.66) (20/24)	29.22 (27.16) (22/25)	0.327
In-Hospital Mortality, *n* (%).	807 (7.89)	29,185 (11.81)	<0.001	71 (20.06)	4284 (25.63)	0.017

**Table 2:** Distribution of study variables and hospital outcomes of patients with and without bronchiectasis hospitalized with community-acquired pneumonia (CAP) in Spain (2016–17), after propensity score matching.

	Bronchiectasis	No Bronchiectasis	*p*-Value
Male Sex, *n* (%)	6204 (60.65)	6220 (60.8)	0.819
Female Sex, *n* (%)	4026 (39.35)	4010 (39.2)	
Age, Mean (SD)	75.47 (13.67)	75.51 (14.33)	0.836
<40, *n* (%)	263 (2.57)	294 (2.87)	0.218
40–64, *n* (%)	1369 (13.38)	1423 (13.91)	
65–74, *n* (%)	2054 (20.08)	1972 (19.28)	
≥75, *n* (%)	6544 (63.97)	6541 (63.94)	
Charlson Comorbidity Index, Mean (SD)	1.02 (0.99)	0.96 (0.92)	<0.001
Acute Myocardial Infarction, *n* (%)	363 (3.55)	310 (3.03)	0.038
Congestive Heart Failure, *n* (%)	1882 (18.4)	1801 (17.61)	0.140
Peripheral Vascular Disease, *n* (%)	678 (6.63)	565 (5.52)	0.001
Cerebrovascular Disease, *n* (%)	512 (5)	434 (4.24)	0.009
Dementia, *n* (%)	498 (4.87)	511 (5)	0.675
Rheumatoid Disease, *n* (%)	397 (3.88)	288 (2.82)	<0.001
Peptic Ulcer, *n* (%)	48 (0.47)	33 (0.32)	0.095
Mild Liver Disease, *n* (%)	546 (5.34)	478 (4.67)	0.029
Type 2 Diabetes Mellitus, *n* (%)	2411 (23.57)	2363 (23.1)	0.428
Hemiplegia or Paraplegia, *n* (%)	46 (0.45)	48 (0.47)	0.836
Renal Disease, *n* (%)	1618 (15.82)	1497 (14.63)	0.019
Cancer, *n* (%)	734 (7.17)	724 (7.08)	0.786
Moderate/Severe Liver Disease, *n* (%)	91 (0.89)	61 (0.6)	0.015
Metastatic Cancer, *n* (%)	213 (2.08)	214 (2.09)	0.961
AIDS, *n* (%)	106 (1.04)	92 (0.9)	0.317
Underwent Surgery, *n* (%)	214 (2.09)	192 (1.88)	0.270
Computerized Axial Tomography of Thorax, *n* (%)	1198 (11.71)	681 (6.66)	<0.001
Bronchial Fibroscopy, *n* (%)	143 (1.4)	101 (0.99)	0.007
Non-Invasive Mechanical Ventilation, *n* (%)	248 (2.42)	198 (1.94)	0.017
Invasive Mechanical ventilation, *n* (%)	150 (1.47)	205 (2)	0.003
Dialysis, *n* (%)	64 (0.63)	87 (0.85)	0.060
Pressure Ulcer, *n* (%)	125 (1.22)	229 (2.24)	<0.001
Length of Hospital Stay, Mean (SD) (Median/Inter Quartile Range)	10.44 (9.64) (8/7)	8.96 (8.52) (7/7)	<0.001
In-Hospital Mortality, *n* (%)	807 (7.89)	1092 (10.67)	<0.001

**Table 3:** Distribution of study variables and hospital outcomes of patients with and without bronchiectasis hospitalized with non-ventilator hospital-acquired pneumonia (NV-HAP) in Spain (2016-17), after propensity score matching.

	Bronchiectasis	No Bronchiectasis	*p*-Value
Male Sex, *n* (%)	242 (68.36)	245 (69.21)	0.808
Female Sex, *n* (%)	112 (31.64)	109 (30.79)	
Age, Mean (SD)	72.44 (18.82)	72.81 (17.99)	0.786
<40, *n* (%)	23 (6.5)	17 (4.8)	0.462
40–64, *n* (%)	50 (14.12)	58 (16.38)	
65–74, *n* (%)	62 (17.51)	72 (20.34)	
≥75, *n* (%)	219 (61.86)	207 (58.47)	
Charlson Comorbidity Index, Mean (SD)	1.35 (1.13)	1.21 (1.08)	0.111
Acute Myocardial Infarction, *n* (%)	17 (4.8)	12 (3.39)	0.343
Congestive Heart Failure, *n* (%)	105 (29.66)	102 (28.81)	0.804
Peripheral Vascular Disease, *n* (%)	27 (7.63)	23 (6.5)	0.557
Cerebrovascular Disease, *n* (%)	35 (9.89)	31 (8.76)	0.605
Dementia, *n* (%)	17 (4.8)	14 (3.95)	0.582
Rheumatoid Disease, *n* (%)	15 (4.24)	10 (2.82)	0.309
Peptic Ulcer, *n* (%)	10 (2.82)	7 (1.98)	0.461
Mild Liver Disease, *n* (%)	27 (7.63)	18 (5.08)	0.166
Type 2 Diabetes Mellitus, *n* (%)	91 (25.71)	89 (25.14)	0.863
Hemiplegia or Paraplegia, *n* (%)	6 (1.69)	4 (1.13)	0.524
Renal Disease, *n* (%)	55 (15.54)	53 (14.97)	0.834
Cancer, *n* (%)	35 (9.89)	32 (9.04)	0.700
Moderate/Severe Liver Disease, *n* (%)	10 (2.82)	10 (2.82)	0.999
Metastatic Cancer, *n* (%)	23 (6.5)	19 (5.37)	0.525
AIDS, *n* (%)	4 (1.13)	6 (1.69)	0.524
Underwent Surgery, *n* (%)	100 (28.25)	102 (28.81)	0.868
Computerized Axial Tomography of Thorax, *n* (%)	59 (0.17)	36 (0.1)	0.011
Bronchial Fibroscopy, *n* (%)	9 (0.03)	4 (0.01)	0.162
Non-Invasive Mechanical Ventilation, *n* (%)	20 (0.06)	10 (0.03)	0.062
Invasive Mechanical Ventilation, *n* (%)	40 (0.11)	46 (0.13)	0.490
Dialysis, *n* (%)	6 (0.02)	11 (0.03)	0.220
Pressure Ulcer, *n* (%)	16 (0.05)	22 (0.06)	0.317
Length of Hospital Stay, Mean (SD) (Median/Inter Quartile Range)	27.05 (25.66) (20/24)	29.58 (28.69) (22/25)	0.321
In-Hospital Mortality, *n* (%)	71 (20.06)	90 (25.42)	0.088

**Table 4:** Multivariable analysis of factors associated with in-hospital mortality during admissions for community-acquired pneumonia (CAP) and non-ventilator hospital-acquired pneumonia (NV-HAP) among patients with bronchiectasis.

	CAP	NV-HAP
	OR (95% CI)	OR (95% CI)
Male Sex	1.09 (0.93–1.28)	1.16 (0.63–2.12)
<40 Years Old	1	1
40–64 Years Old	1.51 (0.52–4.37)	
65–74 Years Old	2.82 (1.01–7.89)	0.22 (0.08–0.64)
≥75 Years Old	6.98 (2.53–19.25)	0.72 (0.35–1.49)
Congestive Heart Failure	1.71 (1.45–2.02)	
Dementia	1.79 (1.37–2.34)	
Cancer	1.53 (1.18–1.99)	
Moderate/Severe Liver Disease	2.26 (1.14–4.49)	
Metastatic Cancer	4.96 (3.46–7.1)	2.87 (1.08–7.61)
Non-Invasive Mechanical Ventilation	2.29 (1.6–3.26)	
Invasive Mechanical Ventilation	5.91 (3.71–9.4)	2.64 (1.14–6.1)
Dialysis	2.44 (1.26–4.72)	9.74 (1.33–71.29)
Pressure Ulcers	3.1 (2.01–4.76)	
*Streptococcus pneumoniae*	0.66 (0.48–0.92)	
*Haemophilus influenzae*	0.09 (0.01–0.61)	
Underwent Surgery	1.66 (1.1–2.49)	

## Supplementary Materials

The following are available online at https://www.mdpi.com/2077-0383/9/8/2339/s1, Table S1: ICD-10 codes for the clinical diagnosis and procedures used in this investigation; Table S2: Distribution of pneumonia pathogens in patients with and without bronchiectasis hospitalized with community-acquired pneumonia (CAP), and non-ventilator hospital-acquired pneumonia (NV-HAP) in Spain (2016-17), after propensity score matching. Table S3: Multivariable analysis of factors associated with in-hospital mortality during admissions for community-acquired pneumonia (CAP), and non-ventilator hospital-acquired pneumonia (NV-HAP) among patients with and without bronchiectasis.
