# Supplementary material for: Hospitalizations for Community-Acquired and Non-Ventilator-Associated Hospital-Acquired Pneumonia in Spain: Influence of the Presence of Bronchiectasis. A Retrospective Database Study
Source: J Clin Med. 2020 Jul 22;9(8):2339. doi: 10.3390/jcm9082339 (PMC7463658; doi:10.3390/jcm9082339)
Supplement: Supplementary file 1 [file jcm-09-02339-s001.pdf]

Supplementary Table S1. ICD-10 codes for the clinical diagnosis and procedures used in this investigation

	**ICD-10 codes**
Acute myocardial infarction	I25.2
Congestive heart failure	I50.x
Pheripheral vascular disease	I71.x, I73.9, Z95.8, Z95.9
Cerebrovascular disease	I69.x
Dementia	F00.x-F02.x
Type 2 Diabetes Mellitus	E11.x
Rheumatoid disease	M05.x, M06.0, M32.x, M33.2, M34.x, M35.3
Peptic ulcer	K25.4-K25.7, K26.4-K26.7, K27.4-K27.7, K28.4-K28.7
Mild liver disease	K70.3, K71.7, K73.x, K74.3-K74.6
Hemiplegia or paraplegia	G81.x, G82.0-G82.2
Renal disease	N18.x
Cancer	C00.x-C26.x, C30.x-C34.x, C37.x-C41x, C45.x-C58.x,C60.x-C76.x, C81.x-C85.x,C88.3, C88.7, C88.9,C90.0, C90.1, C91.x-C93.x, C94.0-C94.3,C94.5, C94.7, C95.x, C96.x,
Moderate/severe liver disease	K72.1, K72.9, K76.6, K76.7
Metastatic cancer	C77.x-C79.x
AIDS	B20.x-B22.x
Computerized axial tomography of thorax	BW24
Bronchial fibroscopy	0BJ08ZZ
No invasive mechanical ventilation	5A09357, 5A09457, 5A09557
Invasive mechanical ventilation	5A1945Z, 5A1955Z, 5A1935Z
Dyalisis	5A1D00Z, 5A1D60Z,3E1M39Z
Pressure ulcers	L89.xxx

Supplementary Table S2. Distribution of pneumonia pathogens in patients with and without bronchiectasis hospitalized with community-acquired pneumonia (CAP), and non-ventilator hospital-acquired pneumonia (NV-HAP) in Spain (2016-17), after propensity score matching.

	CAP		p-value	NV-VAP		p-value
	**Bronchiectasis**	**No bronchiectasis**		**Bronchiectasis**	**No bronchiectasis**	
Streptococcus pneumoniae, n(%)	784(7.66)	870(8.5)	0.027	11(0.03)	9(0.03)	0.650
Klebsiella pneumoniae,n(%)	64(0.63)	40(0.39)	0.018	2(0.01)	3(0.01)	0.654
Haemophilus Influenzae, n(%)	160(1.56)	101(0.99)	<0.001	3(0.01)	1(0)	0.316
Pseudomonas aeruginosa, n(%)	433(4.23)	101(0.99)	<0.001	25(0.07)	12(0.03)	0.028
Non-specified Streptococcus, n(%)	31(0.3)	29(0.28)	0.796	1(0)	0(0)	0.317
Staphylococcus aureus, n(%)	143(1.4)	84(0.82)	<0.001	5(0.01)	12(0.03)	0.086
Escherichia coli, n(%)	93(0.91)	38(0.37)	<0.001	11(0.03)	6(0.02)	0.220
Other Gram negative bacteria, n(%)	128(1.25)	51(0.5)	<0.001	5(0.01)	6(0.02)	0.761
Candidiasis, n(%)	36(0.35)	12(0.12)	0.001	1(0)	2(0.01)	0.563
Aspergillus, n(%)	47(0.46)	5(0.05)	<0.001	5(0.01)	2(0.01)	0.254
Legionella, n(%)	0(0)	5(0.05)	0.025	0(0)	0(0)	NA

Supplementary Table S3. Multivariable analysis of factors associated with in-hospital mortality during admissions for community-acquired pneumonia (CAP), and non-ventilator hospital-acquired pneumonia (NV-HAP) among patients with and without bronchiectasis

.

	CAP	NV-HAP
	OR (95%CI)	OR (95%CI)
Male	1.06(1.03-1.09)	1.03(0.95-1.12)
<40 years old	1	1
40-64 years old	2.01(1.28-3.83)	1.2(0.87-1.424)
65-74 years old	4.32(3.16-6.76)	2.18(1.45-2.91)
> 74 years old	8.9(7.83-10.12)	3.07(2.32-4.24)
Acute myocardial infarction	1.11(1.04-1.17)	
Congestive heart failure	1.4(1.36-1.44)	1.31(1.2-1.43)
Peripheral vascular disease	0.92(0.87-0.98)	
Cerebrovascular disease	1.47(1.4-1.53)	1.34(1.21-1.48)
Dementia	1.95(1.88-2.02)	1.3(1.12-1.51)
Chronic Obstructive Pulmonary	0.67(0.65-0.69)	
Rheumatoid Disease	0.88(0.8-0.96)	
Mild liver disease	1.14(1.07-1.22)	
Type 2 diabetes mellitus	0.87(0.84-0.9)	
Hemiplegia or Paraplegia	1.74(1.55-1.96)	
Renal disease	1.16(1.13-1.2)	1.18(1.07-1.3)
Cancer	2.11(2.02-2.19)	1.62(1.46-1.79)
Moderate/Severe Liver disease	2.79(2.51-3.09)	2.67(2.2-3.23)
Metastatic Cancer	5.75(5.48-6.03)	2.87(2.53-3.26)
AIDS	1.32(1.12-1.55)	3.41(2.25-5.17)
Non Invasive Mechanical ventilation	2.79(2.59-2.99)	1.95(1.67-2.28)
Invasive Mechanical ventilation	8.03(7.51-8.58)	3.3(2.97-3.66)
Dyalisis	2.25(2.03-2.49)	2.16(1.83-2.55)
Pressure ulcers	2.26(2.13-2.4)	1.38(1.18-1.61)
Streptococcus Pneumoniae	0.6(0.56-0.63)	0.54(0.4-0.73)
Haemophilus influenza	0.34(0.27-0.43)	0.52(0.33-0.82)
Surgery	1.07(1.02-1.15)	0.63(0.58-0.68)
Bronquiectasias	0.83(0.76-0.90)	0.87(0.62-1.08)
